# Youth Attitude to Noise Scale (YANS) questionnaire adaptation into Brazilian Portuguese

**DOI:** 10.1016/S1808-8694(15)30484-5

**Published:** 2015-10-19

**Authors:** Angela Maria Fontana Zocoli, Thais Catalani Morata, Jair Mendes Marques

**Affiliations:** 1 Communications Disorders – University of Tuiuti – Paraná, Speech and Hearing Therapy; 2 Communications Disorders – University of Cincinnati, Professor – Graduate Program in communications disorders – Tuiuti University of Paraná, UTP; Researcher at the National Institute for Occupational Safety and Health, Speech and hearing therapist; 3 Geodesic Sciences – Federal University of Paraná and Professor at the Graduate Program in communications disorders – Tuiuti University of Paraná, UTP

**Keywords:** adolescent, hearing, music, noise

## Abstract

The growing exposure of teenagers to environmental noise and music has generated interest in studies about the impact of such exposure, as well as the measures taken in these situations. Therefore, it is fundamental to use a valid and reliable instrument.

**Aim:**

to validate a Brazilian Portuguese version of the Youth Attitude to Noise Scale questionnaire.

**Study design:**

cross-sectional, repeated measures.

**Materials and Methods:**

Translation from English into Portuguese, pre-test, linguistic adaptation, review of grammar and idiomatic equivalence, and translation from Portuguese back to English. Instrument application. Retesting within 30 to 90 days. Measurement of the questions used following Likert's scale. Through factorial analysis, explanation for the connections among a set of variables.

**Results:**

there was a match between translation and counter-translation. The questions were satisfactorily understood. The factorial analysis was well defined with the use of four factors. The instrument's reproducibility was proven by the 0.75 Cronback Alpha general index.

**Conclusions:**

Significant correlations, indicating the construction and content validity for its use, serving as a tool to assess the attitudes of the youth facing exposure to environmental noise or music.

## INTRODUCTION

Today, many leisure activities bring risk to hearing. Such activities include shooting, motor mechanics, motorcycling, going to discos, personal listening devices (MP3 player, MP4 and iPod), rock/pop concerts, home stereo systems (home theater) and automobile stereos. One can not forget the excessive use of music sounds in parties, dances, meetings, cinema, theaters, and also the one generated by street sound cars (“trios elétricos”)^1^.

Although short periods of amplified music do not cause permanent hearing loss, chronic exposure damage are cumulative, in such a way that a mild hearing loss during childhood may eventually become substantial in adulthood^2^.

Noise has become a growing environmental problem in today's western society and has an important role to play in the development of general hearing health problems. Olsen^3^ warns that teeager years are an important stage in life, in which the individual goes through biological, psychological and social changes. During this stage, parental influence is reduced since the very demands and requirements of adolescents increase. They try to find their own life style, change habits, attitudes and behaviors, which can have consequences for their future health.

That is why it is necessary to identify the level of information adolescents have, regardless of growing hearing losses caused by noise exposure during leisure, as well as their own attitudes facing this situation. Therefore, it is necessary to seek the proper instrument to carry out this function. In speech and hearing therapy, more specifically as far as audiology is concerned, there are not too many studies designed to establish what is essential within the adaptation process of a questionnaire^1^.

In order to validate such a questionnaire, Schmidt et al.^4^ stated that only a careful translation is not enough because the linguistic terms must be proper to social and cultural conditions of the population to be studied, there is the need for psychometric measures within a specific context, considering the fact that each society has its own beliefs, habits and attitudes, and its behavior reflects the culture of a country. Whenever considering a questionnaire, language must be clear, simple, however, can not lose its original and coherent equivalence.

It is desirable to have the researcher participate in the adaptation of such a questionnaire because it allows mentioning the explored concepts, reformulating the questions and avoid idiomatic expressions^5^. And, as selecting the questionnaire to be used, the researcher must have in mind the aspects to be approached. Melchiors et al.^6^ state that the questionnaire must be useful not only to identify the individual's behavior at a certain moment in his/her life, but also to establish changes after an educational intervention. They stress that one of the fundamental aspects in creating such a tool is the number of questions and the time needed to answer them, because very long questionnaires are difficult to use.

The use of an questionnaire to assess how young people behave when facing noisy environments, is an important resource to be used by the audiologist in the process of investigating the exposure to high intensity sounds (musical or not), because of the possibility of measuring such behavior. It is a way to know the difficulties individuals have when facing noise and, based on it, be able to implement an educational process. In order to employ it in the academic world, it is necessary to create a Brazilian Portuguese version of it, showing its evaluative properties at an accessible language. Thus, the goal of the present study was to validate the Youth Attitude to Noise Scale – YANS7 to the Brazilian Portuguese Language.

## MATERIALS AND METHODS

245 adolescents from both genders participated in this study (49% were males and 51% females); with ages between 14 and 18 years (mean of 15.7 years); students from high school (24% in their 3rd year; 37% in their 2nd year and 39% in their first year) from a private school in Blumenau, state of Santa Catarina, Brazil.

Initially, the researcher visited the schools in order to talk to the managing teams about the research project. At this time, she handed to them a form asking for permission to carry out the research and a copy of the questionnaire to be used (appendix 1).

After being approved, at the previously established dates and times, the researcher went to the school in order to apply the questionnaire, always with a teacher assigned by the principal. She was then introduced to the students at the beginning of the classes, when she explained her goals with the study and how the students should fill out the questionnaire.

For those students younger than 18 years of age, an informed consent form was sent to their parents, asking for authorization for their child (ren) to participate in the study. In this form the author details the procedures, risks and benefits, responsibility for eventual damage, privacy, as well as the consent intent and study participation for the parents. The document was examined by the Ethics Committee of the institution through protocol # 062/2006, and was approved on 09/14/2006.

The Youth Attitude to Noise Scale (YANS) was developed by Olsen and Erlandsson^7^. Initially with 31 questions that after analyzed, adjusted and selected (taking into account the similarity among the items), were finalized with a total of 19 items. The parameters used to do the factorial analysis revealed the distribution of the questions into four factors, each one involving correlated questions, and they were classified as: attitudes regarding noise associated with the youth's culture; attitudes associated with the skill of being able to concentrate in noisy environments; attitudes concerning day-to-day noises and attitudes to influence the sound environment (Annex 2).

Apendix 1 **brazilian portuguese adapted version Youth behavior as far as noise is concerned (YANS – brazilian)**

1. Eu acho que o volume do som nas discotecas, bailes, shows de rock e eventos esportivos, em geral, é alto demais. F1
2. Ouvir música enquanto faço tarefa escolar ajuda a me concentrar. F2
3. Estou preparado para fazer algo que torne o ambiente escolar mais silencioso. F4
4. Quando o nível de som está muito alto, eu considero a possibilidade de sair de uma discoteca, show de rock, baile ou evento esportivo. F1
5. Consigo me concentrar mesmo se há muitos sons diferentes à minha volta. F2
6. Acho desnecessário utilizar protetor auditivo quando estou numa discoteca, show de rock, baile ou evento esportivo. F1
7. É importante para mim, tornar o som do meu ambiente mais confortável. F4
8. Eu não gosto quando está quieto à minha volta. F2
9. O volume do som em discotecas, bailes, shows de rock ou eventos esportivos, não é um problema. F1
10. Barulhos e sons altos são aspectos naturais de nossa sociedade. F1
11. O barulho do trânsito não é perturbador. F3
12. O nível do som deveria ser diminuído em discotecas, shows de rock, bailes ou eventos esportivos. F1
13. Eu acho que a sala de aula deveria ser silenciosa e calma. F4
14. Os sons de ventiladores, geladeiras, computadores, etc., não me perturbam. F3
15. Eu estou preparado para desistir de atividades onde o volume do som é alto demais. F1
16. O volume do som na minha escola é confortável. F3
17. Para mim, é fácil ignorar barulho de trânsito. F3
18. Deveria haver mais regras ou regulamentos para o volume de sons na sociedade. F1
19. Quando não posso me livrar de sons incômodos, eu me sinto desamparado. F4

Appendix 2 **original version youth attitude to noise scale (YANS)**

1. I think that the sound level at discos, dances, rock concerts and sporting events, in general, is too loud. (F1)
2. Listening to music while doing homework helps me concentrate. (F2)
3. I am prepared to do something to make the school environment quieter. (F4)
4. I consider leaving a disco, rock concert, dance or sporting event if the sound level is too loud. (F1)
5. I can concentrate even if there are many different sounds around me. (F2)
6. I think it is unnecessary to use earplugs when I am at a disco, rock concert, dance or sporting event. (F1)
7. It is important for me to make my sound environment more comfortable. (F4)
8. I don't like when it is quiet around me. (F2)
9. The sound level at discos, dances, rock concerts or sporting events is not a problem. (F1)
10. Noise and loud sounds are natural parts of our society. (F1)
11. Traffic noise is not disturbing. (F3)
12. The sound level should be lowered at discos, rock concerts, dances or sporting events. (F1)
13. I think it should be quiet and calm in the classroom. (F4)
14. Sounds from fans, refrigerators, computers, etc., do not disturb me. (F3)
15. I am prepared to give up activities where the sound level is too loud. (F1)
16. The sound level at my school is comfortable. (F3)
17. It is easy for me to ignore traffic noise. (F3)
18. There should be more rules or regulations for the sound levels in society. (F1)
19. When I cannot get rid of sounds that bother me, I feel helpless. (F4)

The answers are reactions to statements issued through the Likert scale with five grades, where: 1 – “I fully disagree”; 2- “I partially disagree”; 3- “I agree”; 4- “I partially agree”; 5- “I fully agree”.

### Questionnaire validation

The validation process is made up of many stages. Initially, we had a bilingual teacher translate the questionnaire from English into Portuguese. Having the translation, the following step was to take the translated version of the questionnaire for Brazilian field professionals (two speech and hearing therapists and one otolaryngologist), fluent in English, to analyze the document. They did not find significant differences between the translation and the original document, except for some minor differences in verb agreement – justifiable because of the complexity of the Portuguese language.^5^

After the analyses, we randomly selected 30 students from another school in order to pre-test the questionnaire, at a time that would not impair their school activities. Of the thirty students selected, two missed school and one had a hearing disorder, however the latter asked for permission to answer the questionnaire, thus making up a total of 28 students who took part on the study.

The pre-test questionnaire were employed by the researcher at a pre-established time appointed by the school principal and with a duration of 20 minutes, in a coordinated fashion – after the examiner's reading and explanation (about the research objectives and how the answers must be given). The teenagers were asked to answer the questionnaire, record their difficulties in interpretation, their opinion on the language used (whether it was proper and/or if there were any unknown word of expression), and they were also asked to tell us of the difficulties they had to answer it.

The adolescents answered the questionnaire through which the authors investigated their attitudes concerning noise. The adolescents answered the questionnaire in which the authors investigated their attitude regarding noise, personal history and hearing habits. They reported that they did not have difficulties in answering it and the only unknown word for most of them was “stereophonic system”, which was replaced by “stereo” (“home theater” in English). Most of the young people were interested in the study and at the end of the questionnaire; they asked some questions about the comfortable levels of noise.

The semantic (grammar and vocabulary) and cultural equivalence was checked for every item (experiences lived within the cultural context of society), besides the specific care with the instructions to fill it out and the coherence of the presentation. In this specific case, the young people commented that the term currently used to talk about a disco/dancing place was “night out”, answered as being “each and every activity associated to going out (dance place, disco, concert, even going to a bar).

The following stage was to write the final version with the adjustments, which was sent to a second bilingual teacher (without any contact or information about the original version), so that he would do the inverse process of the translation – from Portuguese back into English, and it was revised and compared to the original document by three bilingual teachers in order to check and see whether or not the questionnaire had lost its characteristics.

Of all the adolescents (245) who took part in the study and answered the questionnaire, 50 were randomly selected to answer the same questionnaire again within 30-90 days, in order to conclude the validation process to the Brazilian Portuguese YANS.

### Statistical Analysis

The scope of the questionnaire was analyzed by means of an explosive factor analysis. The aim of the scale used was to explore the behaviors of adolescents towards noise, for dealing with different sounds which are common in the environment where they live. The values assigned to the scales reflect positive or negative associations as far as noise is concerned. The results of this study followed the analysis by factors according to the Olsen's classification^3^. In the calculations they used the scores per questionnaire area and total and these scores were analyzed in relation to gender.

In order to establish the questionnaire's validity, a factorial statistical analysis was carried out; and in order to establish reliability, two techniques were used: the one that compares testing and retesting, and the Cronbach Alpha. In the test-retest comparison we calculated the agreement coefficient between the results by means of the Bland-Altman test and the t-Student paired test in order to check for the existence of differences between the average values of the results.

For all the statistical tests we used a 5% significance level and for the analyses we used the “Sphinx Léxica”, “Analyze-it”, Excel and “Statistica” software.

## RESULTS

The team of specialists which analyzed the translations pointed out that there was a correspondence between the items translated, semantic equivalence between the two translations and no translation difficulty. Adjustments were made to the few differences in verbal. Therefore, the counter-translation compared to the original version did not reveal the need for changes in grammar structure, when the Portuguese version was translated back into English.

The factorial analysis aimed at explaining the correlation between a set of correlated variables, in terms of a small number of latent or non-observable variables. Or, the reduction in data scope to identify a small number of factors which can explain most of the variations observed in a much greater number of variables. The factors were taken in their order of importance, in other words, from the most explanatory to the least explanatory. Table 1 shows the factorial analysis results by the main component method with a four factors solution and Varimax rotation.

**Table 1:** Factorial analysis to define the factors

VARIABLES	FACTORIAL LOADS				
	F1	F2	F3	F4	COMMONALITIES
12	0.824189				0.686191
09	0.772329				0.621483
01	0.727526				0.547602
15	0.677886				0.511794
04	0.625040				0.404890
18	0.566929				0.460222
10	0.379862				0.397013
17		0.770649			0.659752
11		0.673576			0.490741
16		0.472778			0.336225
14		0.472339			0.337206
19		0.406060			0.179038
02			0.698662		0.535373
08			0.670822		0.476530
05			0.528736		0.309930
13			0.511999		0.433394
06				0.634136	0.420377
07				0.473601	0.338712
03				0.461717	0.414310
Explained Variation (%)	18.00	10.60	9.30	7.15	

The factorial analysis was well defined with the use of four factors or four dimensions, and the first factor (F1) was made up of the variables: 12, 9, 1, 15, 4, 18 and 10 which characterize the attitudes of young people towards noise associated to cultural elements; in the second factor (F2) were their attitudes regarding concentration skills in noisy environments through questions 17, 11, 16, 14 and 19; the third factor (F3) encompass the attitudes towards day-to-day noises, questions 2, 8, 5 and 13; while the fourth factor (F4) corresponds to the attitudes that influence the noisy environment, questions: 3, 6 and 7. We must bear in mind that the factor definition is represented by the questions which have the greatest factorial load, in other words, the higher the factorial load value, the greater is the question's explanatory power, or even, the greater the level of understanding for the question.

As to scale reliability, we initially calculated the Cronbach Alfa Coefficient, with the obtained value of a = 0.749917. Since the desirable value is at least 0.70, the result obtained corroborates the scale reliability to be used in studies with Brazilian adolescents.

We also used the paired Student t-Test and the Bland-Altman test in order to check for test-retest reliability. The t-paired Student test calculation (p = 0.41) did not show significant differences between the test-retest result averages and the Bland-Altman test showed a good agreement among the results. These results can be seen on Table 2.

**Table 2:** Test t-paired and agreement test between test-retest

	Mean ± SD		Agreement limits (95%)	Confidence interval (95%)
Test	Retest	Difference (Test- Retest)	Inferior = −2.1	[-2.2; −2.0]
2.9 ± 1.3	2.8 ± 1.5	0.0 ± 1.1	Superior = 2.1	[1.9; 2.2]

The mean scores and standard deviation of the four factors and the entire YANS are shown on Table 3, Table 4 (including the gender variable).

**Table 3:** Factors (f) and the integer yans, mean (m) and standard deviations (SD)

	m	SD
F1 (7 questions) Noise-related attitudes associated with the culture of youth (for example. go to discos).	2.36	1.15
F2 (5 questions) Attitude facing daily noise (for example traffic noises).	2.76	1.29
F3 (4 questions) Attitudes concerning the skill to be able to concentrate in noisy environments.	3.38	1.28
F4 (3 questions) Attitudes to influence the noisy environment (for instance at school).	3.13	1.37
General	2.80	1.31

**Table 4:** Factors and the integer yans: mean and standard deviation according to gender

FACTOR	GENDER	MEAN	STANDARD DEVIATION
F1	Males	2.32	1.15
	Females	2.37	1.14
F2	Males	2.67	1.30
	Females	2.86	1.28
F1	Males	3.26	1.30
	Females	3.50	1.25
F4	Males	3.05	1.40
	Females	3.20	1.33
GENERAL	Males	2.73	1.32
	Females	2.87	1.31

We established the Spearman correlations among the Factors and the entire YANS, noticing that the greatest correlations happen between each factor and the entire YANS, which matches the meaning of the scales. The Cronbach Alpha has a higher value for the F1 factor and decreasing values for the other factors, being consistent for their meaning. The first factor is the one responsible for explaining most of the questionnaire's variability. The attitudes' scale was built taking into account the three quartiles, and the value of the sample scale considered all the way up to the first quartile classified the attitude as negative (25%), between the first and the third quartiles as middle attitude (50%) and from the third quartile and up as a positive attitude (25%).

Table 5 shows the correlations between the factors and the entire YANS, the variation explained for each factor, the Cronbach Alpha and the cutting points for the attitude categories of each factor.

**Table 5:** Correlation between the factors and the integer YANS

	F1	F2	F3	F4	Integer YANS
F1 (Youth culture)	–	–	–	–	–
F2 (Concentration)	0.221	–	–	–	–
F3 (Daily noise)	0.259	0.203	–	–	–
F4 (Intention to influence)	0.327	0.183	0.190	–	–
YANS (Integer)	0.788	0.620	0.570	0.517	–
Cronbach Alpha	0.804892	0.536854	0.496953	0.05515	0.749917
Explained Variation	18.00	10.60	9.30	7.15	
Negative attitude	1.00 – 1.71	1.00 – 2.40	1.00 – 2.75	1.67 – 2.67	1.37 – 2.47
Neutral attitude	1.72 – 2.86	2.41 – 3.20	2.76 – 4.00	2.68 – 3.67	2.48 – 3.11
Positive attitude	2.87 – 4.29	3.21 – 4.60	4.01 – 5.00	3.68 – 4.67	3.12 – 4.21

## DISCUSSION

The questionnaire selected is based on comparative studies carried out in Sweden and in the United States. So far, there was no specific questionnaire, culturally adapted and validated, able to assess the attitudes of adolescents towards noise in Brazil.

Validation is usually a long process which allows one to have a questionnaire which is equivalent to the original one. Through such methodology it is possible to guarantee source equivalence^8^. Based on the adapted questionnaire, it is possible to create the means to evaluate the effects of the educational process regarding noise in leisure activities in Brazil, from the results of attitudes' scale regarding noise, thus helping prevent the damage caused by noise exposure to adolescents.

A questionnaire needs to be reliable, valid and responsive^9^. The reproducibility of this instrument was shown by the general index a = 0.75. The understanding of the questions was satisfactory, because besides the adolescents not having difficulties to answer the questionnaire; the correlations were significant, indicating its construction validity and content. We agree that the aforementioned authors stress that an instrument must be able to lead to an equal result when assessing the same fact under different circumstances, to reliably measure what it intends to assess and be able to detect changes along time or those associated with interventions.

Retesting happened about three months after the test application, showing responses which were very similar among themselves. However, in the retesting we noticed that the attitudes regarding noise, often times were regarded as something negative. This brings back the fact that the repeated application of an instrument after a period of time can determine a change in attitude over a given behavior, since the person can be sensitized by the topic which is being investigated and shows a change which is due only to a previous measuring. Although the test-retest correlations represent a good procedure to measure reliability, there are limitations. A small test-retest correlation may fail to prove low test reliability; however, it can indicate that the very underlying theoretical concept has changed.

The reliability of the Brazilian YANS was proven taking into account that all the correlations were significant, because in all of them there was a p < 0.05 even after 60-90 days, and it can be used in Brazilian young people as an instrument to assess their behavior under exposure to environmental noise.

As we compare the results from the factorial analysis of the present investigation with Olsen's^3^ and Widén's^10^ analysis, we found differences in the distribution of the four factors – as noted below. The four factors found in this study:

Factor 1: Attitudes concerning noise associated with youth's cultural aspects (questions 1, 4, 9, 10, 12, 15, 18);

Factor 2: Attitudes concerning day-to-day noises (questions 11, 14, 16, 17, 19);

Factor 3: Attitudes concerning noise and concentration (questions 2, 5, 08, 13);

Factor 4: Attitudes concerning the skill to influence the sound environment (questions 6, 7, 3).

The four factors defined in Widén's study were.10:

Factor 1: Attitudes concerning noises associated with youth's culture aspects (1, 4, 6, 9, 12, 15, 10, 18);

Factor 2: Attitudes concerning the concentration skill (2, 5, 8);

Factor 3: Attitudes concerning day-to-day noises (11, 14, 16, 17);

Factor 4: Attitudes which influence the sound environment (3, 7, 13, 19).

The four factors described in Olsen's thesis3:

Factor 1: Attitudes concerning noises associated the very culture of youth (1, 12, 9, 15, 6, 4);

Factor 2: Attitudes concerning everyday noises (10, 11, 14, 16, 17);

Factor 3: Attitudes concerning the skill to impact the sound environment (3, 7, 18, 13);

Factor 4: Attitudes concerning noise and concentration (2, 8, 5, 19).

The definition of value is given by the variable which has the greatest factorial load. The factorial analysis establishes the level of understanding for the variables and where they can be better grouped according to the responses collected, even when the studies are from 2004 and 2006 – carried out by the same researchers – results show differences which can be associated to the very teenage culture, considering they encompassed different populations.

As we analyze such study, we can notice that variable 6, for instance, had a greater weight in the factor F4; in the Olsen's study^3^ the greatest weight of the same variable happened on F2 and in the Widén et al.^10^ which was on F1. In factors two, three and four, there were also variable inversions if compared to the results from these authors.

## CONCLUSION

Analyzing the Brazilian Portuguese version of the YANS questionnaire, we notice that it is a valid and reproducible tool to measure the behavior of Brazilian adolescents facing environmental noise and loud music. Such tool may also be used to study behavioral change after an educational intervention.

## References

[1] Zocoli AMF (2007). Hábitos e atitudes de jovens catarinenses frente ao ruído: avaliação com a versão em português do questionário YANS [Dissertação].

[2] Chung JH, Roches Des CM, Meunier J, Eavey RD (2005). Evaluation of Noise-Induced Hearing Loss in Young People Using a Web-Based Survey Technique. Pediatrics..

[3] Olsen SE (2004). Psychological aspects of adolescents' perceptions and habits in noisy environments [Tese].

[4] Schmidt LP, Teixeira VN, Dall'Igna C, Dallagnol D, Smith MM (2006). Adaptação para a língua portuguesa do questionário Tinnitus Handicap Inventory: validade e reprodutibilidade. Rev Bras Otorrinolaringol..

[5] Bradley C, Bradley C (1994). Handbook of Psychology and Diabetes.

[6] Melchiors AC, Correr C, Rossignoli P, Pontarolo R, Fernández-Llimós F (2004). Medidas de avaliação da qualidade de vida em diabetes. Parte II: Instrumentos específicos. Seguim Farmacoter..

[7] Olsen-Widén SE, Erlandsson SI (2004). Self-reported tinnitus and noise sensitivity among adolescents in Sweden. Noise Health..

[8] Torres HC, Hortale VA, Schall VT (2005). Validação dos questionários de conhecimento (DKN-A) e atitude (ATT-19) de Diabetes Mellitus. Rev Saude Publica..

[9] Ferreira CA, Sant'Anna, Cukier A (2006). Avaliando a DPOC pela perspectiva do paciente. J Bras Pneumol..

[10] Widen SE, Holmes AE, Erlandsson SI (2006). Reported Hearing Protection Use in Young Adults from Sweden and the USA: Effects of Attitude and Gender. Int J Audiol..

